# Recurrent Appendicitis in Children: The Impact of a Poorly Known Disease

**DOI:** 10.7759/cureus.46350

**Published:** 2023-10-02

**Authors:** Sara Monteiro, Mariana Capela, Ana Rita Araújo, Marta Tavares, João Pinto

**Affiliations:** 1 Paediatric Department, Centro Materno-Infantil do Norte, Centro Hospitalar Universitário de Santo António, Porto, PRT; 2 Paediatric Department, Hospital Lusíadas Porto, Porto, PRT; 3 Paediatric Allergology Unit, Centro Materno-Infantil do Norte, Centro Hospitalar Universitário de Santo António, Porto, PRT; 4 Paediatric Gastroenterology Unit, Centro Materno-Infantil do Norte, Centro Hospitalar Universitário de Santo António, Porto, PRT; 5 Paediatric Surgery Department, Hospital Escola da Universidade Fernando Pessoa, Porto, PRT; 6 Institute of Research, Innovation and Development, Fundação Fernando Pessoa (FP-I3ID), Porto, PRT; 7 EPI Unit, Instituto de Saúde Pública da Universidade do Porto (ISPUP), Porto, PRT

**Keywords:** recurrent appendicitis, chronic appendicitis, appendicular inflammation, children, abdominal pain

## Abstract

Chronic and recurrent appendicitis is rare in pediatric patients and can be easily misdiagnosed due to its unusual presentation and low incidence rate. We present the case of an 11-year-old male with recurrent right lower quadrant (RLQ) pain persisting for 19 months. The patient experienced pain flare-ups accompanied by paleness and gait limp, without fever or other symptoms. Despite extensive medical examinations, including imaging and endoscopy, a definitive diagnosis remained elusive. As serial abdominal ultrasounds reported an appendix at the upper limit of the normal caliber and symptoms persisted despite medical therapy, a diagnostic laparoscopy was performed, revealing a congested ileocecal appendix with erosions and granulocytic inflammatory infiltrate, consistent with appendicitis. Post-appendectomy, the patient's symptoms resolved, significantly improving his quality of life (QoL), as evidenced by the DISABKIDS Chronic Generic Module (DCGM). This case underscores the challenges in diagnosing chronic and recurrent appendicitis, emphasizing the need for improved awareness, case definitions, and research to better understand and manage these conditions. Moreover, the report highlights the substantial impact of such conditions on patients' physical, social, and psychological well-being using the only health-related QoL instrument developed across cultures for children with chronic diseases: the DCGM.

## Introduction

Recurrent abdominal pain in children is defined as at least three episodes of pain over at least three months, significantly affecting their daily activities. While the majority of cases are considered functional, an organic cause is found in 5% to 10% of cases [1]. There is growing recognition of two distinct and less-understood entities that should be considered in the differential diagnosis of patients with recurrent or chronic right lower quadrant (RLQ) pain: recurrent and chronic appendicitis [2].

Although the definition is not completely consensual, recurrent appendicitis generally involves repeated episodes of acute appendix inflammation over time, with intermittent symptom relief. Chronic appendicitis is marked by persistent, low-grade appendix inflammation, leading to recurring or prolonged abdominal discomfort [3,4].

Literature on recurrent and chronic appendicitis is scarce, with an estimated incidence of up to 6.5% of appendicitis episodes, which may be due to misdiagnosis [5-7].

This paper aims to describe a case of recurrent appendicitis, demonstrating the impact on quality of life (QoL) while increasing awareness of this condition.

## Case presentation

**Figure 1:**
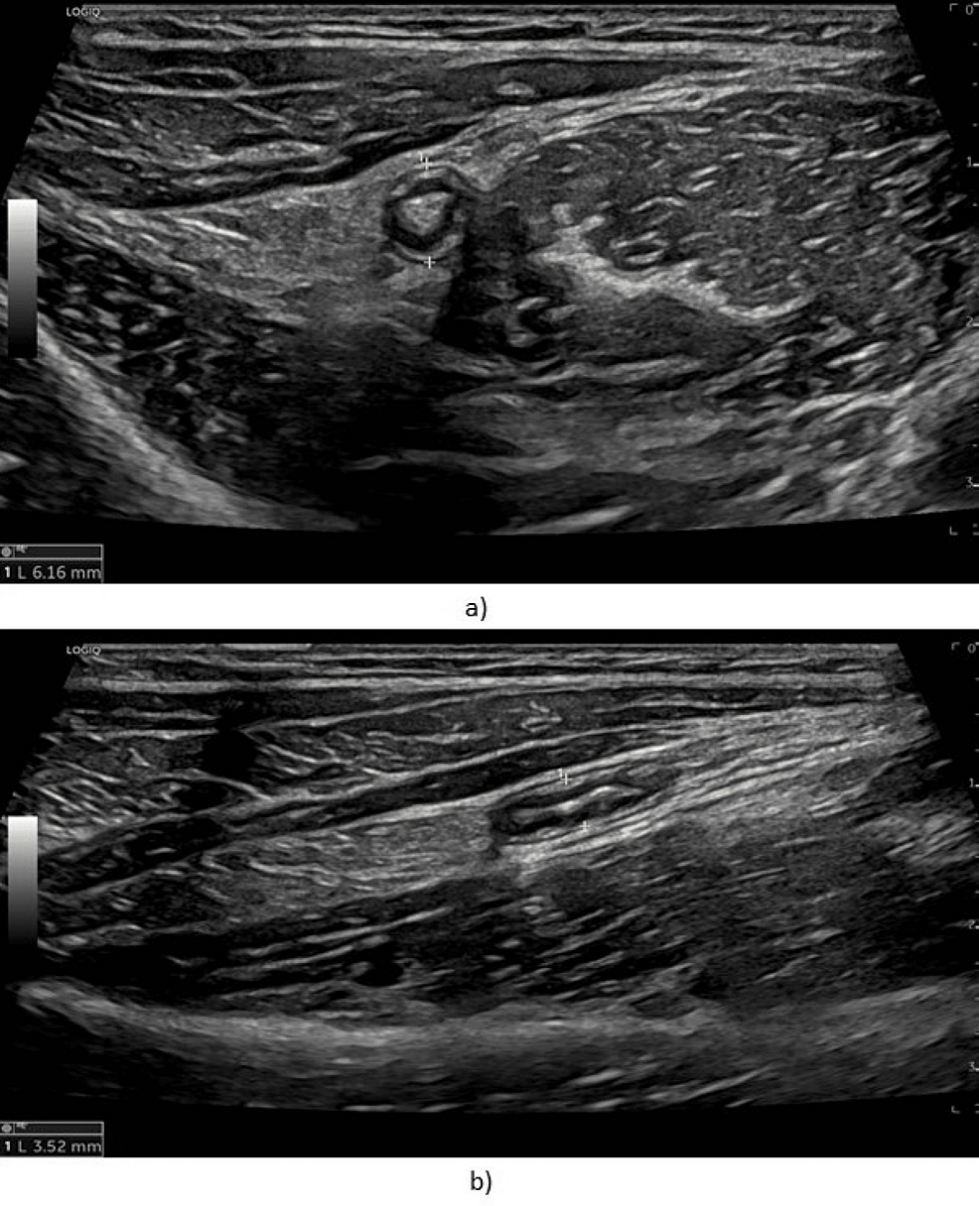
Ultrasound examination showing an appendix with a diameter of 6-7 mm associated with minimal parietal thickening and discrete hyperechogenicity of the adjacent adipose planes (limiting crosses).

An 11-year-old male, healthy except for mild persistent allergic rhinitis to dust mites, reported 19 months of RLQ pain with several outbreaks associated with paleness and gait limp, with an unpredictable duration, lasting hours to several days. There was no fever, digestive problems, or other symptoms, and his stools were normal. At the time of the first visit, he looked sad, and physical examination only revealed RLQ tenderness. Since the symptom’s onset, the patient underwent several exams and treatments. Analytic workup revealed eosinophilia (1160 eosinophils/uL) without increased inflammatory parameters. Parasitological and bacteriological examinations of stools were negative, as well as fecal calprotectin levels. Celiac disease was excluded. Several abdominal ultrasounds reported an appendix at the upper limit of the normal caliber, with nonspecific findings and reactive prominent locoregional ganglionic formations (Figure 1). Abdominal and pelvic magnetic resonance imaging revealed an ileocecal appendix with slightly thickened and enhanced walls, with a maximum caliber of 7 mm, suggesting inflammatory changes, more pronounced in the distal 2/3, with appendicular base spared. He also had a full endoscopic study with biopsies that showed no eosinophilia, Helicobacter pylori infection, or any other changes.

The pain did not improve with analgesia, deworming, or avoidance of cows' milk proteins or wheat. One year after the initial symptoms, he started oral budesonide (9 mg, once daily) with a resolution of pain, which reappeared after weaning. Therapy with amitriptyline, an antidepressant, was also attempted, and it did not improve the patient's symptoms. Regarding antibiotic therapy, metronidazole was administered for five days due to intense aerocolia and suspected dysbiosis.

**Figure 2:**
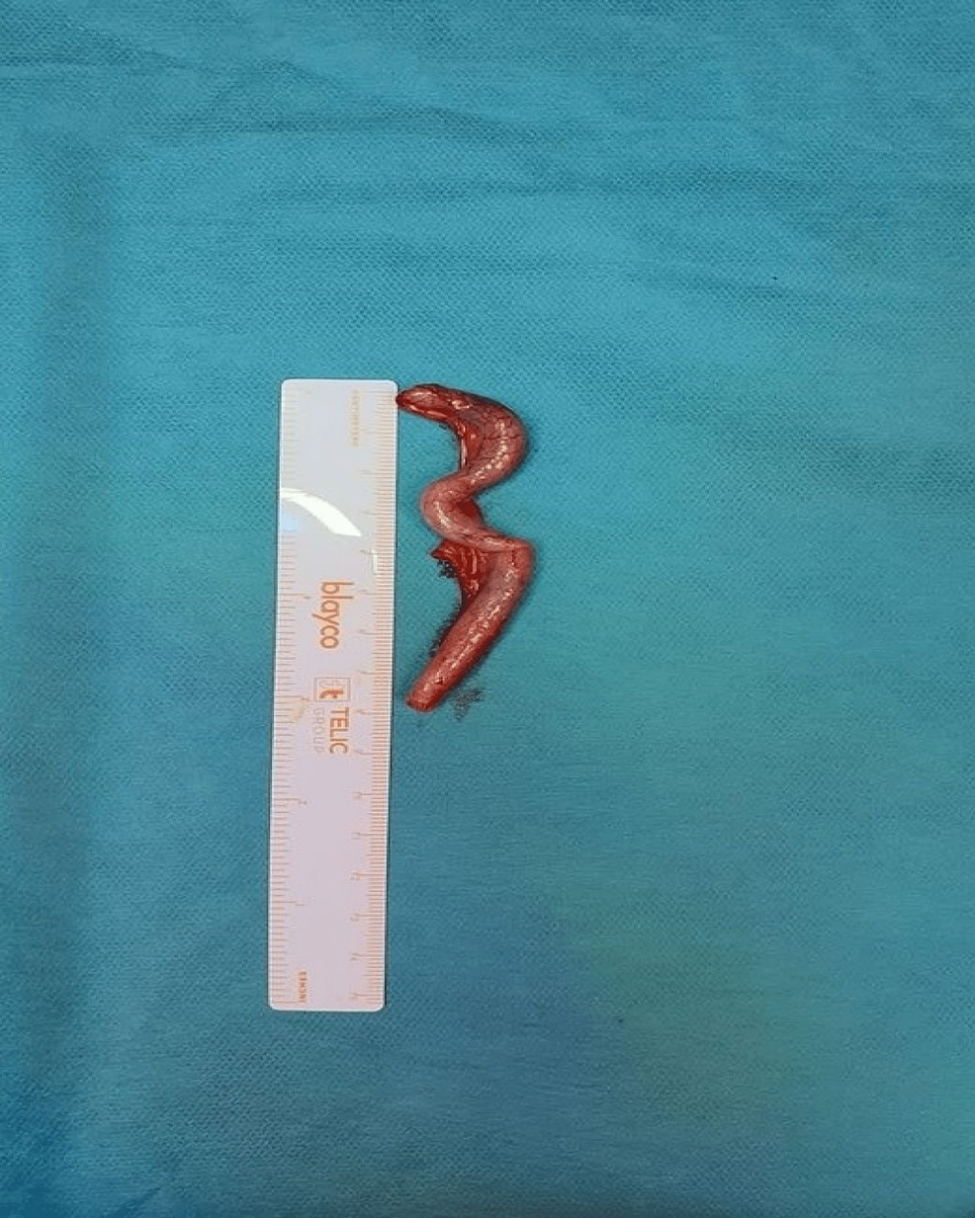
Image showing patient’s appendix after laparoscopic removal.

As abdominal pain persisted and ultrasounds repeated the description of an enlarged appendix, a diagnostic laparoscopy was performed, and the congested ileocecal appendix was removed (Figure 2). The anatomopathological examination revealed erosions and scaly aspects of the mucosa with granulocytic inflammatory infiltrate, limited to the lamina propria, associated with lymphoid hyperplasia; these were suggestive of appendicitis.

Follow-up

He had serial assessments in the first six months after surgery, being completely asymptomatic. Physical examination was unremarkable, he was happy, without any pain or discomfort in the RLQ.

Impact on daily living activities

He had always been a good student, well-integrated in school, enjoying classes and peers. After the onset of the disease, he started skipping school (>3 days per month), and, due to the worsening of the pain, in the three months before the appointment, he missed several days, having attended only two days in the previous one and having lowered his academic results.

Regarding physical activity, he stopped practicing breakdancing and tennis as well as general exercise and gymnastics at school. Although he had not lost weight and maintained his appetite, during periods of pain flare-ups, he refused to eat. He also had a restrictive diet with no improvement in his condition. Sleep was also affected, as he had episodic nocturnal pain that often woke him up.

He described baseline pain with an intensity of 7/10 (severe pain) on the numeric rating scale, 10/10 during flare-ups, and 0/10 (no pain) right after appendectomy. To better assess the impact on QoL, we asked the patient and his parents to complete the 37-item DISABKIDS Chronic Generic Module (DCGM-37), wherein higher scores indicated better QoL. The most affected domain, considered by both parents and patient, was the mental one, with a score of 33 and 23 (minimum of 13 and maximum of 65), respectively. This is followed by the physical (parents score 37 vs. patient score 33) and the social one (parents score 38 vs. patient score 37). The parents’ total score was 108 pre-surgery and the patient’s 93; after surgery, both scored 185 (minimum of 37 and maximum of 185).

## Discussion

Our patient fulfilled the criteria of recurrent abdominal pain and presented as red flags prolonged pain with night waking, localization of pain away from the central abdominal region, and outbreaks with paleness and gait limp. In this sense, the main organic causes of recurrent abdominal pain were progressively excluded, namely, parasitic and bacterial intestinal infections, namely Helicobacter pylori; celiac disease; eosinophilic gastrointestinal diseases (EGIDs); and inflammatory bowel disease [8]. Moreover, it should be noted that this patient did not meet the Rome IV criteria [9] for the diagnosis of functional gastrointestinal disorders, which was corroborated by the lack of response to amitriptyline, which generally improves symptoms, even in children [10].

Although chronic appendicitis and recurrent appendicitis appear controversial, in the past few decades, numerous case reports and case series have emerged, offering substantiating evidence that, although uncommon, they do occur, and should be considered in the differential diagnosis of recurrent abdominal pain [2]. A recent study even advocates that laparoscopic appendectomy should be early considered for patients with unremarkable workup with chronic RLQ abdominal pain of unclear etiology [11].

The terms "chronic" and "recurrent" appendicitis are often used interchangeably. However, at least one source distinguishes them, with the first being characterized as the presence of continuous RLQ pain lasting for three or more weeks and, the second one, as repeated episodes of similar RLQ pain occurring in a sequential manner. Considering this, we classified our case as “recurrent.” Frequently, these patients have a normal white blood cell count and/or a presentation without fever [6,12], which is in agreement with our patient, who only presented peripheral eosinophilia, justified by its known allergic rhinitis.

Chronic appendicitis usually presents with chronic inflammation or fibrosis of the appendix; when clinical recurrent episodes of abdominal pain are combined with histology suggestive of inflammation but without features of chronic appendicitis, it is better called recurrent [13]. Nevertheless, it is worth mentioning that although abdominal pain is attributed to infectious or inflammatory appendiceal pathology, there is no clearly defined histopathological correlation [11]. In our case, the pathology report revealed mild inflammation. However, the patient had been under budesonide for a few months, which acts on the terminal ileum and proximal colon [14] and may explain less exuberant inflammation. The resolution of symptoms after appendectomy supports the diagnosis [15,16]. The histopathological finding of lymphoid hyperplasia, described in our patient, was found to be a predictor of chronic abdominal pain resolution post-appendectomy [11].

The need to exclude other pathologies led to multiple exams, some of them invasive, dietary restrictions, and various therapies. Although not considered a surgical emergency, it is important to be aware of complications that are similar to those of acute appendicitis [17,18].

Furthermore, the impact on patients' QoL was well established in this paper. To our knowledge, this is the first case report that has objectively assessed it. The scores obtained in the DCGM-37 [19] demonstrate the impact at a physical, social, and, above all, psychological level, with a full recovery in all domains after surgery.

## Conclusions

This paper draws attention to a pathology that is still little recognized and probably underdiagnosed, encouraging the report of more cases and research in this field. A better case definition is crucial for an early diagnosis and treatment, minimizing the greater impact on the QoL of these patients.
